# Multimode quantum states with single photons carrying orbital angular momentum

**DOI:** 10.1038/s41598-017-03239-1

**Published:** 2017-06-15

**Authors:** Xin-Bing Song, Shi-Yao Fu, Xiong Zhang, Zhen-Wei Yang, Qiang Zeng, Chunqing Gao, Xiangdong Zhang

**Affiliations:** 1 Beijing Key Laboratory of Nanophotonics & Ultrafine Optoelectronic Systems, School of Physics, Beijing Institute of Technology, Beijing, 100081 China; 2 School of Opto-Electronics, Beijing Institute of Technology, Beijing, 100081 China

## Abstract

We propose and experimentally demonstrate a scheme for generating multimode quantum states with single photons carrying orbital angular momentum (OAM). Various quantum states have been realized by superposing multiple OAM modes of single photons in two possible paths. These quantum states exhibit NOON-like “super-resolving” interference behavior for the multiple OAM modes of single photons. Compared with the NOON states using many photons, these states are not only easily prepared, but also robust to photon losses. They may find potential applications in quantum optical communication and recognizing defects or objects. The method to identify a particular kind of defect has been demonstrated both theoretically and experimentally.

## Introduction

Quantum entanglement plays a key role in quantum information processing such as quantum teleportation, quantum cryptographic, quantum metrology and parallel computing^1–11^. One kind of multiphoton entangled states, known as NOON states, are a particularly useful class of states^11–16^. They contain N indistinguishable particles in an equal-weighted superposition of all being in one of two possible modes *w* and *v*, which are expressed as $\frac{1}{\sqrt{2}}({|N,0\rangle }_{w,v}+{|0,N\rangle }_{w,v})$, and can be used to obtain a high measurement precision that scales as N. Many schemes for the generation of NOON-states have been proposed theoretically^12–18^. However, experimentally, it is extremely challenging. So far, NOON states with only a few photons or particles have been reported experimentally^19–26^. In addition, N-photon entangled states become increasingly sensitive to losses as N grows^27, 28^. In the presence of losses or other types of noise, no two-mode quantum state can beat the standard limit by more than just a constant factor in the limit of large N^29^.

Recently, single-photon NOON-like quantum states $\frac{1}{\sqrt{2}}({|1\rangle }_{R,-2q}+{|1\rangle }_{L,2q})$, superpositions of eigenstates of light with opposite total angular momentum quantum numbers at different polarization modes (right- and left-circular), have been proposed and demonstrated experimentally^30^. Such states in the single-photon regime can be exploited to measure rotation angles with a precision scaling up to *m* times the square root of the number of probes used. Although these states are also affected by angular momentum decoherence such as noises, compared with the N-photon NOON states, the OAM scheme still shows some advantages for the ultra-sensitive angular measurement, because every photon in this regime is disentangled from all others and hence the loss of a photon does not affect the overall phase coherence^30^.

Inspired by the above studies, in this work we propose a kind of new NOON-like quantum states based on single photons with orbital angular momentum (OAM)^31–33^. We call them multimode NOON-like states. In our scheme, multiple OAM modes of single photons have been used to superpose in two possible paths and various NOON-like quantum states can be realized. When the multiple modes degenerate into single modes, our scheme is the same to that in ref. 30. Because the transmission capacity of information can be increased by adding modes, in the past few years many investigations have been done to increase the number of modes in various multiplexers for optical communications^34–36^. Since various multimode NOON-like states can be generated by our scheme, it is expected to have potential applications in optical communications.

## Results and Discussion

### Experiment demonstration of multimode NOON-like states

**Figure 1:**
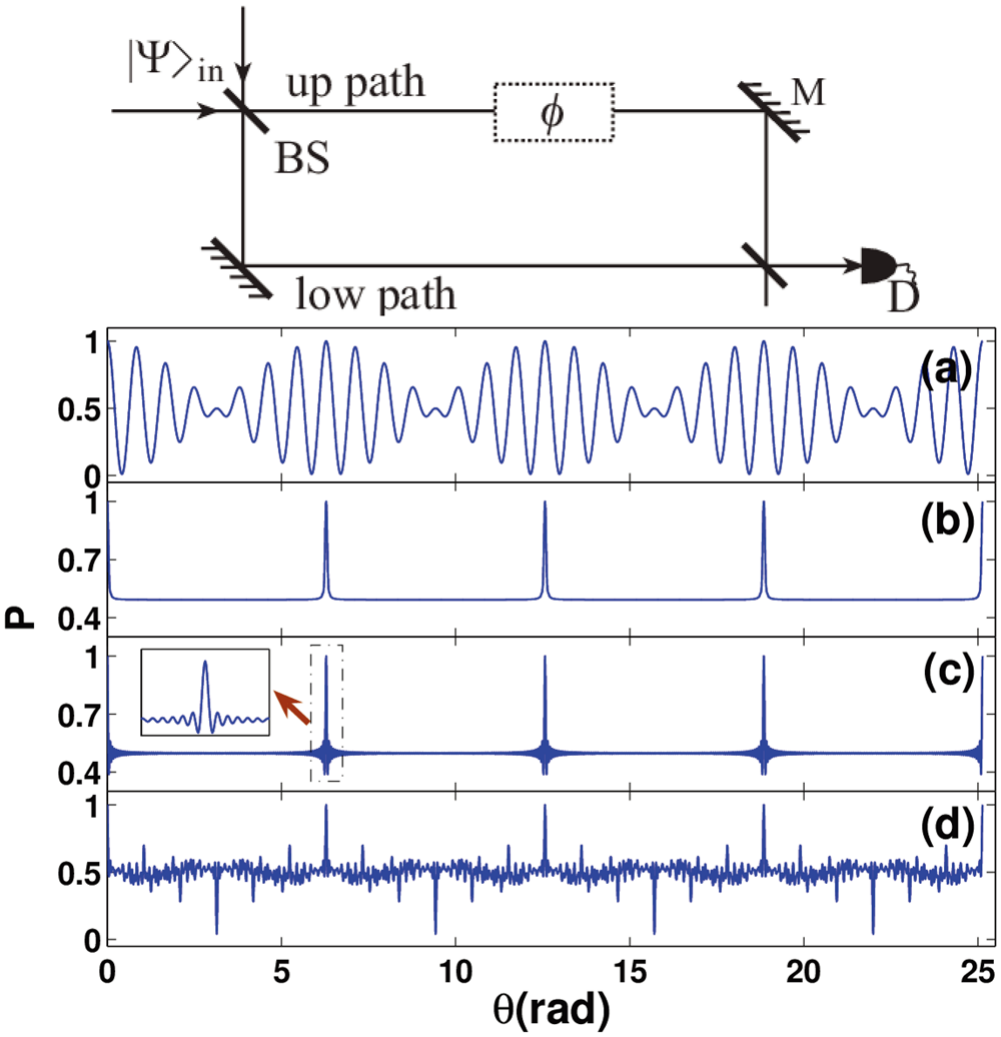
The probability *P* to get the photon by a detector on an output of the interferometer. (**a**) The result for an equivalent mode $L$ composed of equal weighted superposition of ${l}_{7}$ and ${l}_{8}$. (**b**) The result for a mode composed of eigen modes from 1 to 100 with weighting factors ${c}_{p}=101-p$. (**c**) The result for a mode composed of equal weighted OAM eigenmodes from 1 to 100. The inset is the magnification of the box area. (**d**) The result for a mode composed of all prime number modes between 1 and 100 with equal weight. Scheme to test NOON states is shown on top of the figure. ${|{\rm{\Psi }}\rangle }_{in}$ describes a single photon state with superposition OAMs. BS: beam splitter; M: mirror.

We consider that a single photon with OAMs impinges into a Mach-Zehnder (MZ) interferometer as shown on the top of Fig. 1. The two paths are denoted by modes *a* and *b*, respectively. The state of an incident single photon can be written as $\,{{\rm{\Psi }}}_{in}=|L\rangle$, where $L=\frac{1}{\sqrt{D(L)}}\sum _{p}{c}_{p}{l}_{p}$ describes an equivalent OAM mode, $D(L)$ is a coefficient for normalization, and *c*_*p*_ is the probability amplitude of the eigen-mode *l*_*p*_ with *p* being the topological charge. The single photon with such a vortex charge can be obtained by the diffraction of a specially designed hologram^37, 38^. After a beam splitter (BS) and a phase difference *ϕ*, the incident state of the single photon turns to be:

1 $$|\phi {\rangle }_{La,b}=\frac{1}{\sqrt{2}}(|L,{0}_{a,b}\rangle +{e}^{i\varphi }|0,{L}_{a,b}\rangle ),$$

where ${|L,0\rangle }_{a,b}=|{L}_{a}\rangle |{0}_{b}\rangle$ and ${|0,L\rangle }_{a,b}={|0\rangle }_{a}{|L\rangle }_{b}$. ${|L\rangle }_{a}$ (${|L\rangle }_{b}$) represents the OAM $L$ mode corresponding to the path mode $a(b)$, and $|0$ is an empty mode. The state described by Eq. (1) indicates a photon with a superposition of the OAM mode $L$ either in one path or the other. Similar to the relation between the phase and photon number in NOON states, here the phase $\varphi$ is sensitive to OAM eigen-modes. So, Eq. (1) can be further written as

2 $${|\phi \rangle }_{La,b}=\frac{1}{\sqrt{2{\sum }_{p}{|{c}_{p}|}^{2}}}\sum _{p}{c}_{p}({|{l}_{p},0\rangle }_{a,b}+{e}^{ip\theta }{|0,l\rangle }_{pa,b}),$$

where $p\theta$ is the phase for the OAM eigen-mode ${l}_{p}$, and $\theta$ is an angular. Because of orthogonality $\langle {l}_{p}|{l}_{q}\rangle ={\delta }_{pq}$, where $\delta$ is Kronecker function, we can obtain the probability to get the photon without mode discrimination:

3 $$p=\frac{1}{2{\sum }_{p}{|{c}_{p}|}^{2}}\sum _{p}{|{c}_{p}|}^{2}[1+\,\cos ({l}_{p}\theta )].$$

Such a result originates from the superposition of multiple OAM modes, the mode amplitude ${c}_{p}$ can be taken as arbitrary value by controlling a specially designed hologram, which is different from the single mode case described in ref. 30. In addition, a superposition of one photon without the OAM in two paths has been discussed in the previous investigations^39, 40^, which is different from the present case with multiple OAM modes. According to the present design, we can obtain various NOON-like quantum states. For example, if we take two OAM modes ${l}_{7}$ ($p=7$) and ${l}_{8}$ ($p=8$), that is $L=\frac{1}{\sqrt{2}}({l}_{7}+{l}_{8})$, the output NOON-like states exhibit beat wave-like pattern as shown in Fig. 1(a). If $L=\sum _{p=1}^{100}(101-p){l}_{p}/\sqrt{\sum _{p=1}^{100}{(101-p)}^{2}}$ is taken, comb-like pattern appears as shown in Fig. 1(b). Furthermore, if we modulate the weighting factor equaled, that is $L=\frac{1}{\sqrt{100}}\sum _{p=1}^{100}{l}_{p}$, we still get a comb-like pattern as shown in Fig. 1(c). Comparing it with that in Fig. 1(b), a cluster of oscillation appears in such a case, which is shown in the inset of Fig. 1(c). It is interesting that the noise-like pattern as shown in Fig. 1(d) can also be produced, if we choose all prime number of OAM modes between 1 and 100 with the same weight. Recent experiments have shown that the OAM with ${l}_{p} < 300$ can be well modulated^41, 42^. In fact, within such a range, very rich multimode NOON-like states can be constructed.

**Figure 2:**
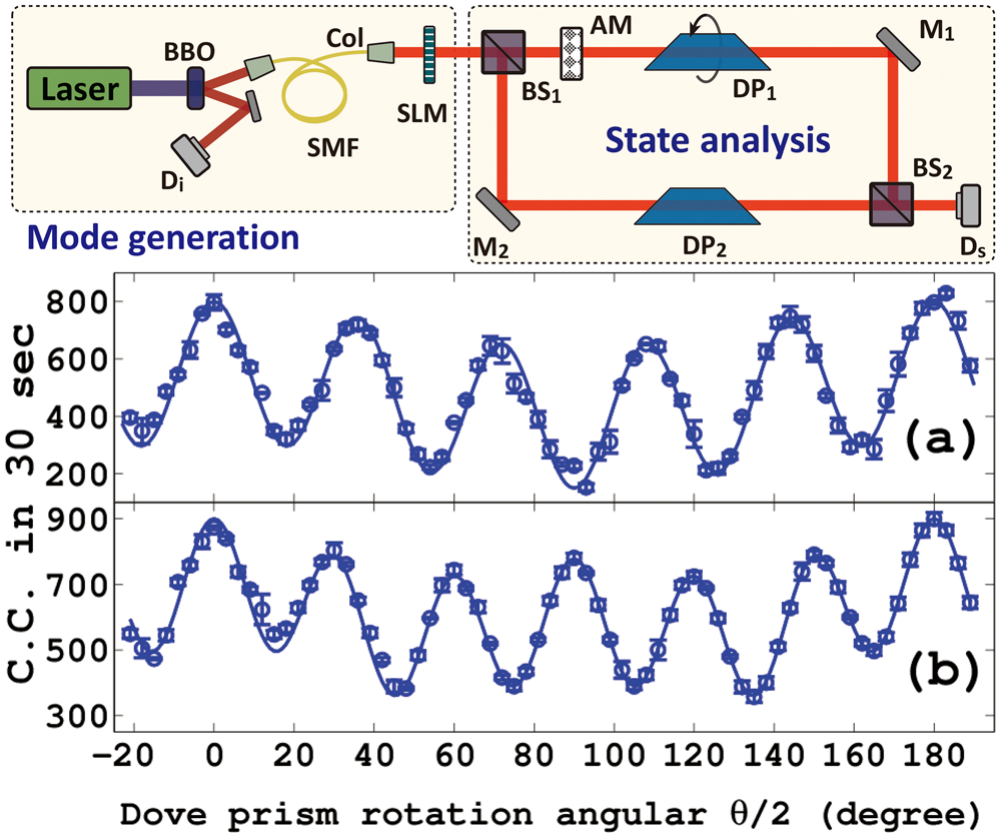
Experimental setup and results for multimode NOON states. SMF: single mode fiber; Col: collimator; SLM: spatial light modulator; AM: absorbing material; BS_1,2_: beam splitter; DP_1,2_: Dove prism; M_1,2_: mirror; D_i,s_: detector. (**a**) and (**b**) show the results for $L={l}_{1}+2.5{l}_{5}$ and $L={l}_{1}+{l}_{2}+2.5{l}_{6}$, respectively. The round dots and solid lines represent the experimental results and theoretical fitting, respectively.

The above theoretical scheme can be realized by the experimental setup as shown on the top of Fig. 2. The scheme consists of two parts: Mode generation and State analysis. The single photon source is provided by the signal photons of entangled photon pairs which are produced through spontaneous parametric down conversion process in a $5\times 5\times 3\,mm$ type-I phase matching beta-barium-borate crystal (BBO). The crystal is pumped by the second harmonic of a Ti:sapphire picoseconds laser (Tsunami, Spectral Physics) with center wavelength 390 nm, and repetition rate 80 MHz. The signal photons are collected into a single mode fiber and projected onto a spatial light modulator (SLM) to gain OAM modes, then pass through an interferometer to reach detector D_s_. The idle photons are detected by D_i_ as a trigger. Both the signal and idler photons are spectrally filtered by interference filters with 10 nm bandwidth centered at 780 nm before arriving at single-photon detectors (Perkin-Elmer SPCMAQR-14). A time window of 5 ns is chosen to capture coincidence counting, and the coincidence rates are recorded as experimental data.

Here we take $L={l}_{1}+2.5{l}_{5}$ and $L={l}_{1}+{l}_{2}+2.5{l}_{6}$ as examples to show how this setup can generate multimode NOON-like states. The choice of these examples is only to facilitate the realization of the experiment without losing generality. First, we manipulate the SLM to generate single photons with OAM modes. Dp_1_ will be rotated in the up path and Dp_2_ is fixed in the low path to compensate path difference. The experimental results for the coincidence counting (circle dots) are shown in Fig. 2(a) and (b) under the condition of pump power 50 mW. The error bars demonstrate experimental statistical deviation. For all plots, error bars are calculated directly from experimental data by repeating measurement, and the instability of interferometer under the finite experimental condition is the key factor. Figure 2(a) corresponds to the case with $L={l}_{1}+2.5{l}_{5}$(${c}_{1}=1$ and ${c}_{5}=2.5$), and Fig. 2(b) to that with $L={l}_{1}+{l}_{2}+2.5{l}_{6}$ (${c}_{1}={c}_{2}=1$ and ${c}_{6}=2.5$). The solid lines in Fig. 2(a) and (b) are the corresponding theoretical results as depicted by Eq. (3). It is seen clearly that the agreements between the experimental measurements and theoretical results are very well. In fact, if we only take one OAM mode such as the topological charge $l$, our results naturally degenerate into the single mode case. In such a case, Eq. (1) can be written as:

4 $${|\phi l\rangle }_{a,b}=\frac{1}{\sqrt{2}}({|l,0\rangle }_{a,b}+{e}^{il\theta }{|0,l\rangle }_{a,b}).$$

The probability to get the photon in one output port is expressed as

5 $$P=\frac{1}{2}[1+\,\cos (l\theta )].$$

**Figure 3:**
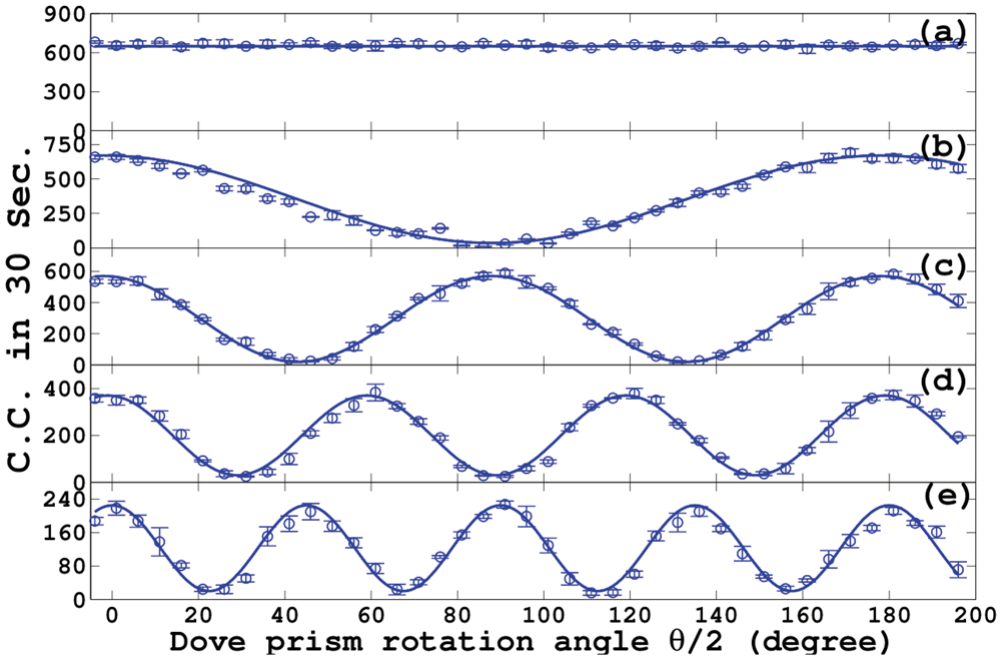
Experimental results for NOON-like states. (**a**) The experimental result for $l=0$. Under the same conditions as in (**a**), the results for $l=1,2,3,4$ are shown in (**b**), (**c**), (**d**) and (**e**), respectively. The round dots and solid lines represent the experimental and theoretical results, respectively.

The single-photon NOON-like quantum states with the single OAM mode have been analyzed in ref. 30. The present method is different from them where the polarization degree of freedom (DOF) is replaced by the path DOF. However, the results are similar. The experimental result for $l=0$ is shown in Fig. 3(a) as a calibration. Under the same conditions, the measured results for $l=1,2,3,4$ are given in Fig. 3(b)–(e), respectively. The circle dots represent the experimental results and the solid lines are corresponding normalized theoretical results. The $l$-fold improvement for the angular measurement precision is observed clearly.

For the scheme in ref. 30, the stability of interferometer is not needed and a polarization measurement gives information on a physical rotation of the reference frame by using a single mode OAM state. However, in the present scheme the stability of interferometer is very important, the generation of multimode quantum states depends directly on it. Based on such a scheme, various multimode quantum states can be realized. Because these quantum states can exhibit NOON-like “super-resolving” interference behavior, they are expected to have some special applications, such as recognition of complex defects or objects.

### Recognition of spiral defects with single photons

The multiphoton NOON states are usually used to obtain high-precision phase measurements. The present multimode NOON-like quantum states we have constructed can not only be applied to this aspect, but we believe they can be applied to optical communication to improve the efficiency of information transfer because of multiple modes involved. Especially, it can be applied to recognition of complex spiral defects or objects. In general, defects always exist in real materials. How to identify defects is an important issue. There are various types of defects, in which the spiral (screw) defects are also very common in the process of fabricating microstructures. The characteristics of these defects have been described in refs 43 and 44. In the following, we discuss how to identify the spiral defects by using these states. When the photon is incident on the material with the defect, the scattered light will be affected by the defect. If the defect can destroy the photons of certain modes (say $q$) with a probability ${\eta }_{q}$, that is, the operator of destroying process can be described by $1-\sum _{q}{\eta }_{q}|{l}_{q}\rangle \langle {l}_{q}|$, when the material with a defect is put in one path (say the up path) of the interferometer on the top of Fig. 2, Eq. (2) becomes

6 $${|{\phi }_{L}\rangle }_{a,b}=\frac{1}{\sqrt{2{\sum }_{p}^{\,}|{c}_{p}{|}^{2}}}[\sum _{p}{c}_{p}({|{l}_{p},0\rangle }_{a,b}+{e}^{ip\theta }{|0,{l}_{p}\rangle }_{a,b})-\sum _{q}{c}_{q}{\eta }_{q}{e}^{iq\theta }{|0,{l}_{q}\rangle }_{a,b}],$$

where the last term in Eq. (6) represents the effect of the defect. Here we assume that the introduction of defects does not affect the coherent superposition, thus, Eq. (6) is still described with a pure state. In such a case, the corresponding probability is

7 $${P}_{b}=\frac{1}{2{\sum }_{p}^{\,}|{c}_{p}{|}^{2}}\{\sum _{p}|{c}_{p}{|}^{2}[1+\,\cos ({l}_{p}\theta )]+\frac{1}{2}\sum _{q}|{c}_{q}{|}^{2}[{{\eta }_{q}}^{2}-2{\eta }_{q}-2{\eta }_{q}\,\cos ({l}_{q}\theta )]\}.$$

We perform the experiments with and without the defect, separately. The difference of the output signals is expressed as

8 $${P}_{\bigtriangleup }=-\frac{1}{4{\sum }_{p}{|{c}_{p}|}^{2}}\sum _{q}{|{c}_{q}|}^{2}[{\eta }_{q}^{2}-2{\eta }_{q}-2{\eta }_{q}\,\cos ({l}_{q}\theta )].$$

**Figure 4:**
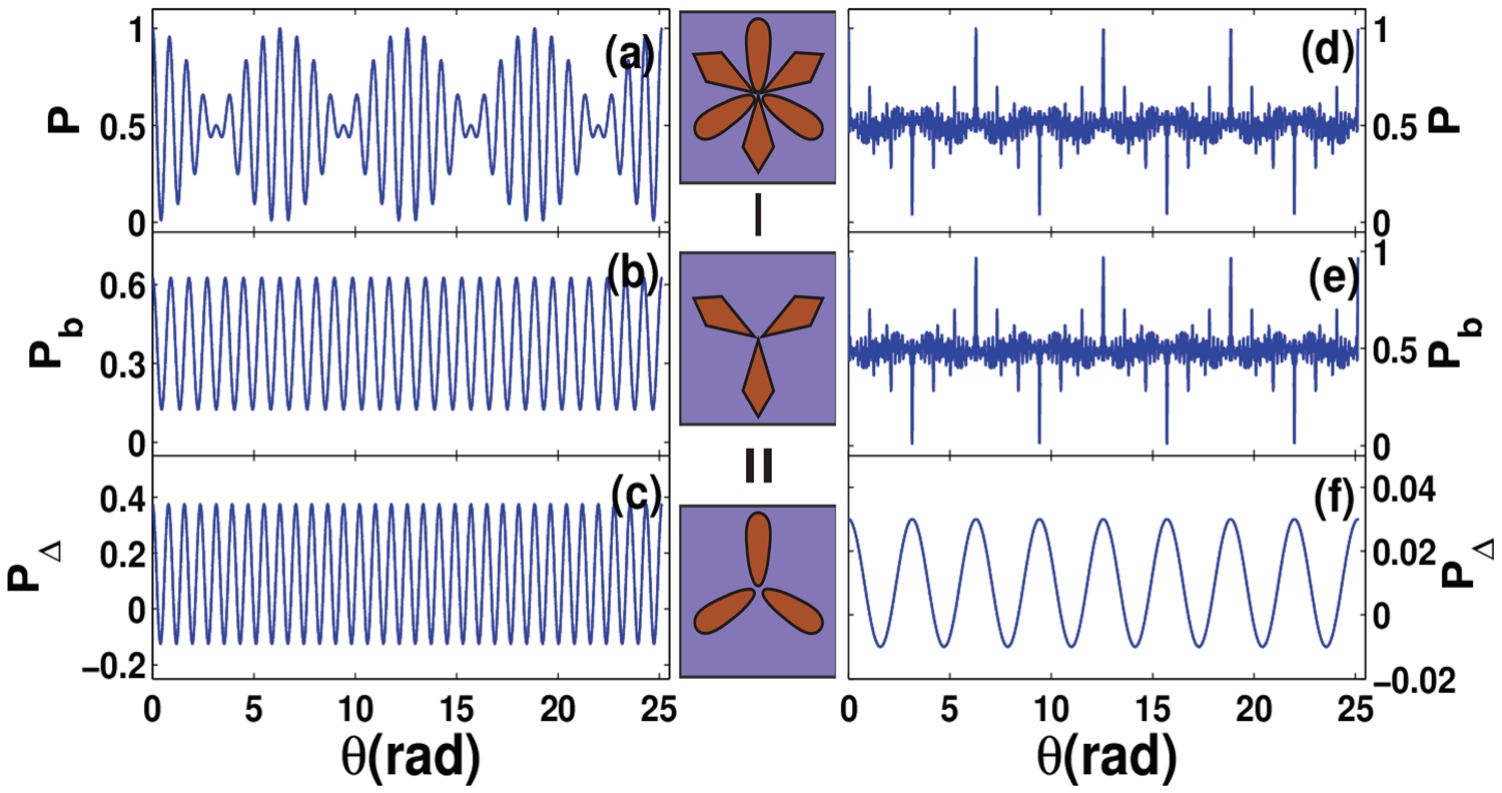
Theoretical results for defect recognition. The center column shows the progress of defect recognition sketch. The top pattern shows the incident multimode NOON states. The middle one is the output pattern when the defect is introduced. The bottom one shows the difference between the above two. The left column displays results for the situation of equal weighted superposition by ${l}_{7}$ and ${l}_{8}$. (**a**) Is the result when ${l}_{7}$ and ${l}_{8}$ are both included, and it is the same as Fig. 1(a). (**b**) Is the result when the mode ${l}_{8}$ is broken. (**c**) Shows the difference between (**a**) and (**b**). The right column shows results for the situation of the mode composed of all prime number modes between 1 and 100 with the equal weight. (**d**) Is the result when all prime modes are included, and it is the same as Fig. 1(d). (**e**) Is the result when the mode ${l}_{2}$ is broken. (**f**) Shows the difference between (**a**) and (**c**).

From Eq. (8), we can get the information of the defect. In the middle column of Fig. 4, we show the defect recognition process vividly. The top pattern shows the multimode NOON-like state and the middle one describes the output result when the defect is introduced. From the difference between them, we can obtain the information of the defect as shown by the bottom pattern. In the left column of Fig. 4, a concrete example is given. In such a case, the incident multimode NOON-like state is taken as the beat wave-like pattern ($L=\frac{1}{\sqrt{2}}({l}_{7}+{l}_{8})$) as shown in Fig. 1(a). The output pattern is given in Fig. 4(b), which describes the interference result of the unaffected mode ${l}_{8}$. The difference between them ($P-{P}_{b}$) is the defect state ${l}_{7}$, which has been shown in Fig. 4(c). If we take the defect mode ${l}_{2}$, and the noise-like multimode NOON-like state (Fig. 1(d)) as the incident state, similar recognition process has also been demonstrated, which is shown in the right column of Fig. 4.

**Figure 5:**
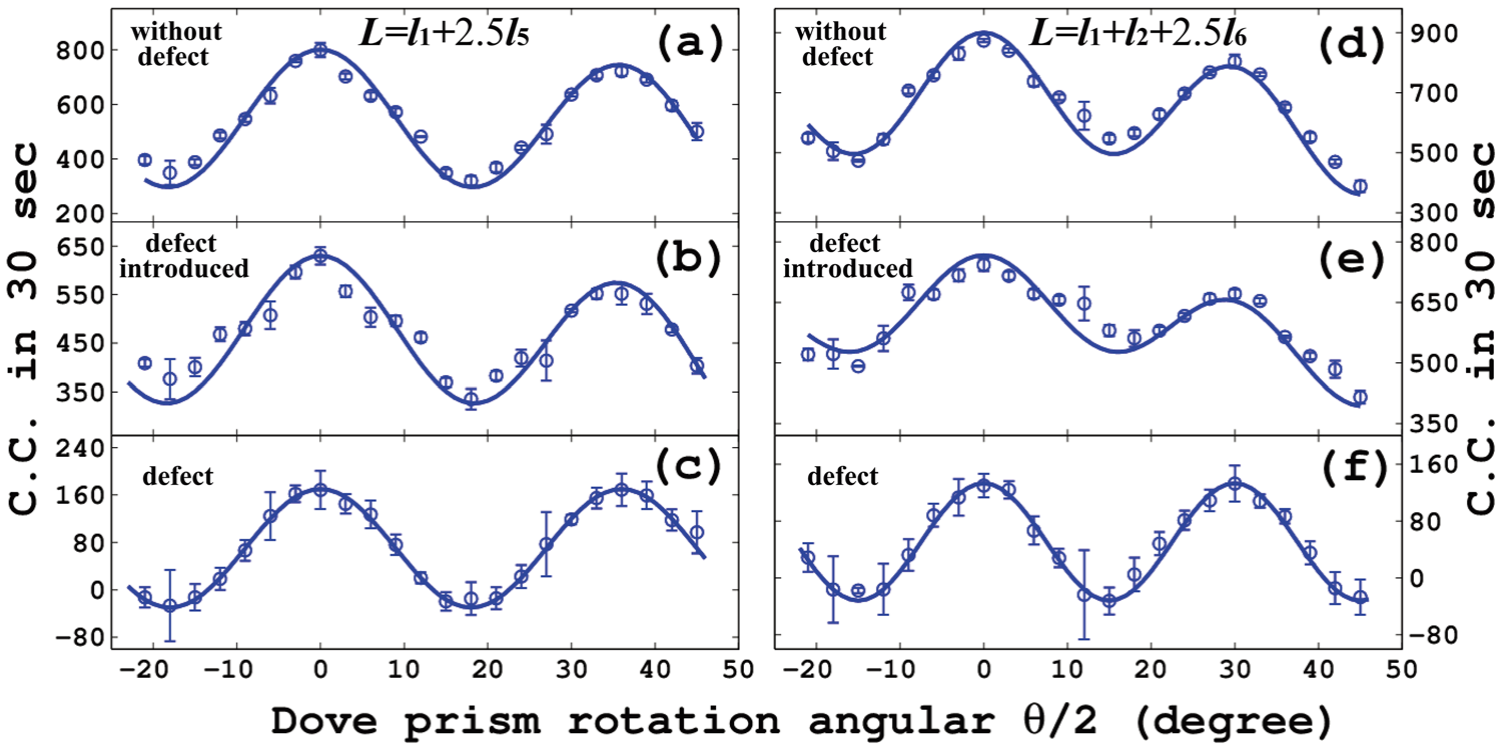
Experimental results for recognition. The left column describes results for the incident NOON state with $L={l}_{1}+2.5{l}_{5}$. (**a**) Is the result when the iris is open and it is part of Fig. 2(a). (**b**) Is the result when the iris is partly closed to block the mode ${l}_{5}$. (**c**) Shows the difference between (**a**) and (**b**). The right column shows results for the incident NOON state with $L={l}_{1}+{l}_{2}+2.5{l}_{6}$. (**d**) Is the result when the iris is open and it is part of Fig. 2(b). (**e**) Is the result when the iris is partly closed to block the mode ${l}_{6}$. (**f**) Shows the difference between (**d**) and (**e**). The round dots and solid lines represent the experimental and theoretical results, respectively.

The defect recognition process can be performed by the experimental setup as shown on the top of Fig. 2. In the experiment, based on the partially space separation of different modes, we use an iris to mimic the defect. This scheme is universally valid for all kinds of OAM modes, but here we just take two examples. One is that the incident multimode NOON state is taken as that described in Fig. 2(a) ($L={l}_{1}+2.5{l}_{5}$), the iris is set to block the photons with an OAM mode ${l}_{5}$, and the results are shown in Fig. 5(a), (b) and (c). The other is that the multimode NOON state described in Fig. 2(b) ($L={l}_{1}+{l}_{2}+2.5{l}_{6}$) has been used as the incident state, the iris is partly closed to block the mode ${l}_{6}$, and the corresponding results are given in Fig. 5(d), (e) and (f). In the experiment, we modulate the weights of ${l}_{5}$ and ${l}_{6}$ bigger than others to make the experiment easier. The round dots and solid lines represent the experimental and theoretical results, respectively. The agreement between the theoretical results and experimental measurements is observed again. We have to point out that the pinhole is unable to discriminate two adjacent modes, here we only provide a demonstration of the principle.

It is worth noting that the sensing processes shown in Figs 4 and 5 work only if the defect objects are present in the querying light. In fact, if the objects are not in the querying light, we can measure the scattered fields of the defect objects as the output signals. In such a case, it is directly related to the quantum radar^45, 46^. In recent years, quantum illumination and radar without OAM photons have been discussed in detail^45, 46^. For the case with OAM photons, the discussions will be given elsewhere. At end, we would like to point out that the present scheme is also applicable to the case of classical OAM beam although the above discussions only focused on quantum states with single photons carrying OAM.

## Conclusions

In summary, we have demonstrated both theoretically and experimentally the generation of multimode NOON-like quantum states using the superposition of OAM multiple modes of single photons in two possible paths. Various NOON-like quantum states have been realized, which is able to return to the single mode case when one OAM mode is taken^30^. This means that NOON-like quantum states with required modes can be designed according to our scheme. Compared with the NOON states using many photons, these states are not only easily prepared, but also robust to photon losses. Thus, the difficulty using many photons can also be avoided. We anticipate that potential applications of the present scheme in optical communications can improve the efficiency of information transfer. It is also anticipated to have important applications in recognition of defects or objects. Based on these NOON-like quantum states, we have also demonstrated both theoretically and experimentally recognition of the particular kind of defect.
